# Hypoxia induces a lipogenic cancer cell phenotype via HIF1α-dependent and -independent pathways

**DOI:** 10.18632/oncotarget.3058

**Published:** 2014-12-11

**Authors:** Alessandro Valli, Miguel Rodriguez, Loukas Moutsianas, Roman Fischer, Vita Fedele, Hong-Lei Huang, Ruud Van Stiphout, Dylan Jones, Michael Mccarthy, Maria Vinaxia, Kaori Igarashi, Maya Sato, Tomoyoshi Soga, Francesca Buffa, James Mccullagh, Oscar Yanes, Adrian Harris, Benedikt Kessler

**Affiliations:** ^1^ Weatherall Institute of Molecular Medicine, University of Oxford, Oxford, UK; ^2^ Target Discovery Institute, Nuffield Department of Medicine, University of Oxford, Oxford, UK; ^3^ Centre for Omic Sciences, Rovira i Virgili University, Reus, Spain; ^4^ Biomedical Research Centre in Diabetes and Associated Metabolic Disorders, Madrid, Spain; ^5^ The Wellcome Trust Centre for Human Genetics, Nuffield Department of Medicine, Oxford, UK; ^6^ Institute for Advanced Biosciences, Keio University, Tsuruoka, Yamagata, Japan; ^7^ Mass Spectrometry Research Facility CRL, Department of Chemistry, University of Oxford, Oxford, UK

**Keywords:** cancer metabolism, fatty acids, HIF1α, HIF2α, hypoxia, Kennedy pathway, lipidomics, PAF

## Abstract

The biochemistry of cancer cells diverges significantly from normal cells as a result of a comprehensive reprogramming of metabolic pathways. A major factor influencing cancer metabolism is hypoxia, which is mediated by HIF1α and HIF2α. HIF1α represents one of the principal regulators of metabolism and energetic balance in cancer cells through its regulation of glycolysis, glycogen synthesis, Krebs cycle and the pentose phosphate shunt. However, less is known about the role of HIF1α in modulating lipid metabolism. Lipids serve cancer cells to provide molecules acting as oncogenic signals, energetic reserve, precursors for new membrane synthesis and to balance redox biological reactions. To study the role of HIF1α in these processes, we used HCT116 colorectal cancer cells expressing endogenous HIF1α and cells in which the *hif1α* gene was deleted to characterize HIF1α-dependent and independent effects on hypoxia regulated lipid metabolites. Untargeted metabolomics integrated with proteomics revealed that hypoxia induced many changes in lipids metabolites. Enzymatic steps in fatty acid synthesis and the Kennedy pathway were modified in a HIF1α-dependent fashion. Palmitate, stearate, PLD3 and PAFC16 were regulated in a HIF-independent manner. Our results demonstrate the impact of hypoxia on lipid metabolites, of which a distinct subset is regulated by HIF1α.

## INTRODUCTION

Reprogramming of metabolism is necessary for cancer cells to sustain their growth and survival under adverse micro-environmental conditions [1-3]. This involves both catabolic and anabolic processes including glycolysis, glutamine dependent anaplerosis, glycogenolysis, amino acid synthesis, nucleic acid synthesis [4-6] and also lipid metabolism [7-9]. Colon adenocarcinoma cells, compared to normal cells, were shown to have a higher number of lipid droplets, a common mechanism used by cells to store triacylglycerides and cholesterol derivatives, thus suggesting an alteration of cancer cells in lipid metabolism towards a lipogenic phenotype [10]. Remodeling of lipid species promoting tumorigenic properties was shown also in ovarian patient tumor tissue [11]. Integrative proteomics and metabolomics analysis demonstrated KIAA1363 (an acetyl monoalkylglycerol ether hydrolase) to be highly elevated in ovarian cancer cells and to regulate lipid signaling including platelet activating factor (PAF) catabolism [12]. In addition, monoacylglycerol-lipase, mediating the hydrolysis of monoacyglycerols (MAG) and thus the intracellular rate of free fatty acid (FAs) production, was highly elevated in cancer cell lines classified as more aggressive [11]. FAs are constituents of MAG, di and tri-acylglycerols (DAG and TAG) and glycerol phospholipids. Prior to FA biosynthesis, the ATP-dependent acetyl-CoA carboxylase biotin-dependent enzyme (ACC) catalyzes the formation of malonyl-CoA by carboxylation of acetyl-CoA. Acetyl-CoA and malonyl-CoA are then condensed by fatty acid synthase (FASN). Seven reaction cycles in sequence, condensing and reducing the added acetyl-CoA, yield palmitate, the most common C16 (C16:0) of the saturated FAs [9]. Palmitate is the precursor of many other FAs as it can go through further elongation/desaturation reactions and thereby provide the diversity of FAs derivatives. The *de novo* FAs synthesis activity in tumor cells was observed nearly 50 years ago [13], contrasting more recent studies that adult cells mostly acquire FAs from dietary sources and rarely use the *de novo* pathway [14]. Consistent with this, OA-519 was identified in breast carcinomas, correlating with FASN activity and poor patient prognosis [15]. The importance of *de novo* FAs synthesis has also been documented in many cancer types, e.g. ovarian and colorectal cancers [16-18]. FASN inhibition diminishes cell proliferation, cell viability and reduces *in vivo* tumor growth [7, 19]. This lipogenic phenotype provides substrates allowing cancer cells to synthetize new cell membranes [8], to store energy and to generate molecules involved in the regulation of cell signal transduction and cell motility, such as lipids rafts, blebs and invadopodia [20-22].

Hypoxia, a hallmark of tumors, triggers pro-lipogenic metabolism mediated by the activity of oncogenic pathways [9]. In hypoxic cancer cells, activation of Akt resulted in an up-regulation of ATP citrate lyase, the enzyme producing the cytosolic pool of the acetyl-CoA substrate of FASN [23, 24]. Also, hypoxia regulates both *in vitro* and *in vivo* FASN expression in human breast tumors through a mechanism involving Akt and HIF1α [23], and recently Ras and hypoxia were shown to play a role in elongation and desaturation of FAs for lipogenesis [25].

HIF-1α is a major regulator of cancer metabolism, particularly glycolysis, glycogen synthesis, TCA cycle, flux into the PPP shunt, nucleotides, amino acids and leptin metabolism [26-29]. However, less is known about the role of HIF in modulating lipid metabolites. We therefore used colorectal cancer cells with the HIF1α gene either deleted or HIF1α and/or HIF2α knocked down to evaluate the effect of HIF1α on lipid metabolites [30]. Our untargeted metabolomics approach including ^1^H-NMR, LC/MS and GC/MS integrated with proteomics, revealed an interplay between HIF1α-dependent and HIF1α-independent alterations of key lipid metabolites and associated enzymes.

## RESULTS

### Hypoxic response of cancer cells and cancer cell lipid phenotype

**Figure 1:**
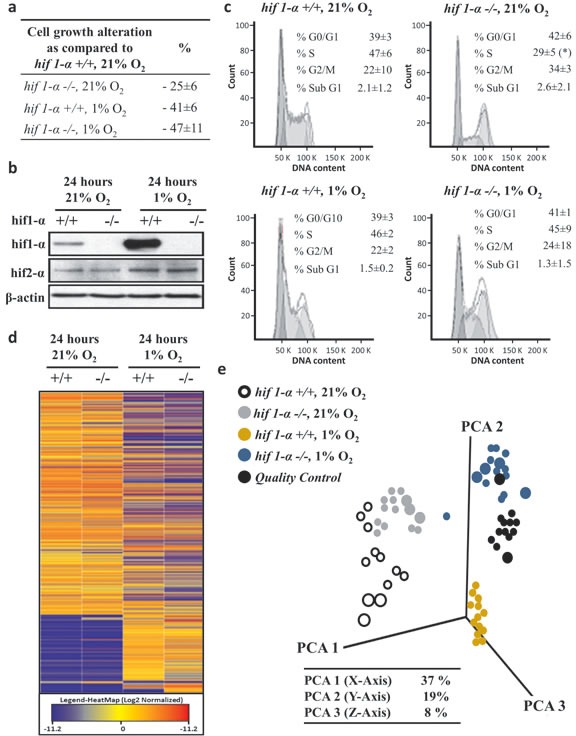
Hypoxia-dependent lipophilic molecular features phenotype of HCT116 colorectal cancer cells (a) Biological triplicates of logarithmically growing HCT116 colorectal cancer cells were plated in equal numbers in 21% O_2_ and 1% O_2_ and collected after 24 hours with a confluence below 85%. Cell numbers are given as a percentage ±sd relative to the number of HIF1α wild type cells in normoxia, set as 100%. (b) HCT116 cells were treated as described above, HIF1α and HIF2α levels detected by western blot analysis induction in hypoxic. HIF1α increased about eight-fold in wild type hypoxic cells compared to normoxia, and no signal was observed in *hif1α^−/−^* cells. HIF2α did not show any compensatory induction in the *hif1α^−/−^* cells. (c) In addition, the percentage ±sd of treated cells in G0/G1, S and G2/M phase of the cell cycle is indicated. Representative DNA profiles are presented. HCT116 cells wild type and *hif1α^−/−^*, normoxic and hypoxic cells were cytofluorometrically investigated for the percentage ±sd of apoptotic cells with sub-G1 DNA content. (d) Heatmap plotting log2 of average relative intensities of 1,487 detected and baseline normalized untargeted molecular features selected with a fold change ≥2 (in at least one group) and a *p*<0.05. Untargeted analysis was conducted by LC/MS QTOF nanoflow on the molecular features in the lipophilic phase with positive mode [+] acquisition. (e) Three dimension Principal Component Analysis plotting the data matrix obtained from the selected 1,487 untargeted molecular features. PCA analysis revealed a differential regulation of molecular features in 1% O_2_ and dependency on the presence of HIF1α (n=5). Percentage of coverage of three principal components analysis is reported. Quality controls were obtained by pooling equal amounts of the analyzed samples, were injected during the analysis at fixed intervals, formed an intermediate cluster assessing the repeatability within the analysis (n=5).

Oxygen tension in solid tumors varies considerably between 0.1–2%. In order to reflect this, we chose 1% as the oxygen concentration in our study. Cell proliferation, given as a percentage ±sd relative to the number of HCT116 HIF1α wild type cells in normoxia, was set as 100%. There was a 25%±6% (*p*-value<0.05) reduction of proliferation observed for HIF1α knockout HCT116 cells (*hif1α^−/−^*) at normoxic levels (Figure 1a). Under hypoxic conditions, proliferation of both wild type cells *hif1α^−/−^*cells was reduced by 41%±6% (*p*-value < 0.05) and 47%±11% (*p*-value<0.01), respectively.

As expected, exposure to 1% O_2_ tension for 24 hours led to HIF1α accumulation in wild type HCT116 cells, while no HIF1α signal was observed in *hif1α^−/−^* cells in normoxic or hypoxic conditions (Figure 1b). The expression of the HIF2α isoform in response to hypoxia was doubled from baseline in both wild type and *hif1α^−/−^* cells, thereby showing no notable compensation of HIF2α levels in the absence of HIF1α (Figure 1b). HIF1α suppression was also observed in DLD-1 and SW1222 HIF1α knock down (*hif1α^KD^*) cell lines (figure S1). Wild type and HIF2α knock down (*hif2α^KD^*) in the DLD-1 cell line showed comparable basal levels of HIF1α under normoxic conditions. HIF1α knock down was confirmed in the double HIF1/2α knock down (*hif1/2α^KD^*) DLD-1 cell line by a two-fold reduction of its observed levels in hypoxia (figure S2). Similarly, HIF2α knockdown levels were comparable when knocked down either alone or in combination with HIF1α both under normoxia and hypoxia conditions. HCT116 wild-type and *hif1α^−/−^* cells did not show any significant difference in cell diameter or volume. However, HCT116 *hif1α^−/−^* normoxic cells showed a significant reduction in progression to S phase as compared to the other conditions tested, and no difference in other cell cycle phases (G0/G1, G2/M and sub G1) were observed (Figure 1c).

Having established the above experimental conditions, a nano-liquid chromatography mass spectrometry (LC/MS) based untargeted metabolomics screen was performed to analyze metabolites in cell extracts derived from wild type and *hif1α^−/−^* HCT116 cells under normoxic and hypoxic conditions resolved by C18 reversed phase chromatography in positive electrospray ionization (ESI+) mode. After the application of a cut off of ≥2 change (in at least one group) with *p*<0.05, 1,487 molecular features were selected in each of the four experimental groups. A comparable profile was observed between wild type and *hif1α^−/−^* cells in normoxia as shown by heatmap and PCA analyses (Figure 1d and e). Interestingly, a clear difference was noted between hypoxic wild type and *hif1α^−/−^* cells, which both differed markedly from the cognate normoxic controls. Finally, quality controls (QCs) profile samples demonstrated repeatability of the nanoflow LC/MS analysis as shown by PCA analysis (Figure 1d and e).

### Classification of the metabolic responses

**Figure 2:**
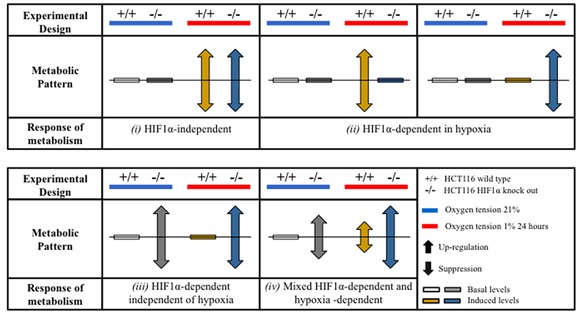
Patterns of adaptation of hypoxic cell metabolism Proteo-metabolomics hypoxic response of colorectal cancer cells in normoxia and hypoxia was classified according to two way ANOVA test of significance of the source of variation (HIF1-α, O_2_ tension and their interaction) and for the statistical significance within the groups after Bonferroni multiple comparisons post-test analysis.

For data analysis purposes, the metabolic effects were classified as shown in a schematic representation (Figure 2): *(i) HIF1α-independent response:* comparable changes (gain or suppression) were seen in wild type and *hif1α^−/−^* cells under hypoxia; *(ii) HIF1α-dependent response in hypoxia:* changes (gain or suppression) were observed in wild type *vs hif1α^−/−^* hypoxic cells only; *(iii) HIF1α-dependent response independent of hypoxia:* the metabolic response was similarly regulated (gain or suppression) in *hif1α^−/−^* vs wild type in normoxic and hypoxic cells; and *(iv) HIF1α-dependent and hypoxia-dependent mixed-response:* absence of HIF1α (*hif1α^−/−^*) caused a regulation (gain or suppression) as compared to wild type cells and hypoxia caused a regulation (gain or suppression) as compared to normoxic cells (Figure 2).

### Characterization of *de novo* FAs biosynthesis in hypoxia

**Figure 3:**
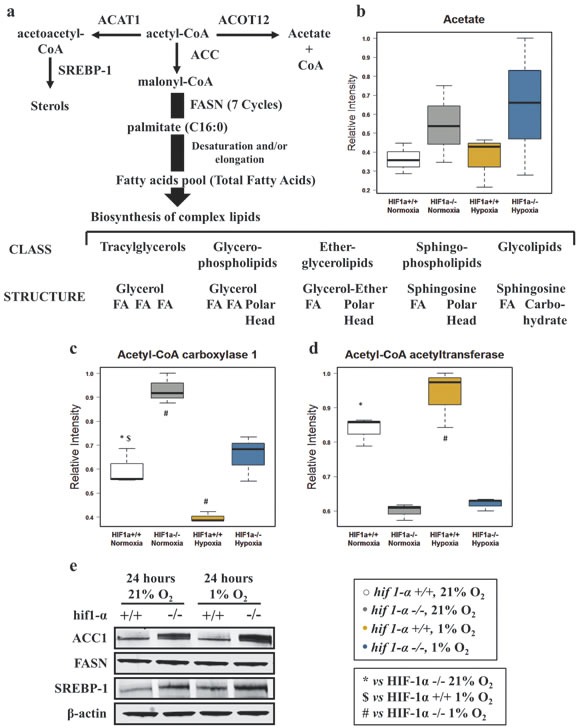
Alteration of key metabolic enzymes for the de novo fatty acids biosynthesis in HCT116 colorectal cancer cells in hypoxia (a) Metabolic pathways of acetyl-CoA forming sterol, fatty acid synthesis or acetate, and enzymes involved. FASN mediated de novo fatty acids biosynthesis precedes the formation of the main classes of complex lipids. (b) Acetate (product of acetyl-CoA catabolism) was detected by ^1^H-NMR in the aqueous phase. Intensities are shown as normalized relative intensities and reported as mean ±sd (n=3). (c and d) Label-free quantitative proteomics analysis reveals altered levels of key metabolic enzymes: Acetyl-CoA carboxylase 1 and Acetyl-CoA acetyltransferase 1. Data are shown as mean ±sd of normalized intensities (n=3). (e) Western blot analysis was used to confirm the pattern of regulation of ACC1. Western blot analysis assessing the hypoxic response of FASN and SREBP-1 in wild type and *hif1α^−/−^*, HCT116 colorectal cancer cells (n=3).

We first examined substrates of the *de novo* FAs biosynthesis and sterol formation. Acetate, the metabolic product of acetyl-CoA, did not show any notable differences in abundance (Figure 3a and b; Table 1). To investigate the regulation of selected enzymes initiating the *de novo* FAs biosynthesis, we performed a label-free quantitative (LFQ) proteomics analysis of HCT116 wild type and *hif1α^−/−^* cells under normoxic and hypoxic conditions. We detected and identified a total of 3,632 proteins at a 1% false discovery rate (FDR). 1,609 proteins were quantified with p≤0.05 within technical replicates. ACC1 levels were evaluated to examine the first committed step of the *de novo* FAs biosynthesis (Figure 3a). Interestingly, hypoxia suppressed ACC1 protein levels in wild type and *hif1α^−/−^* cells as compared to the parental normoxic counterparts. The presence of HIF1α reduced ACC1 levels in both normoxic and hypoxic wild type cells as compared to *hif1α^−/−^*, a result that was confirmed by western blot analysis (Figure 3c and e; Table 2). Acetyl-CoA acetyltransferase 1 (ACAT1), condensing two molecules of acetyl-CoA to acetoacetyl-CoA, was examined to evaluate any effects on ketogenesis as an alternative metabolic pathway to FAs biosynthesis (Figure 3a). ACAT1 levels significantly decreased in the absence of HIF1α in both normoxic and hypoxic *hif1α^−/−^* cells (Figure 3d; Table 2). We also evaluated FASN and SREBP-1 as they are main regulators of FAs synthesis and sterol formation. We observed no changes in FASN levels within our experiments (Figure 3a). A significant accumulation of SREBP-1 levels in normoxia and hypoxia was observed for *hif1α^−/−^* as compared to wild types cells. Hypoxia showed also a mild increase of SREBP-1 in both wild type and *hif1α^−/−^* cells as compared to normoxic cells, thus showing a HIF1α-dependent suppression of SREBP-1 levels in wild type cells (Figure 3e).

**Table 1:** Analysis of lipid metabolites intensities performed by two way ANOVA Statistical significance is shown for the source of variation (HIF1-α, O_2_ tension or their interaction); the statistical significance within the groups is shown after Bonferroni multiple comparisons post-test analysis. Abbreviations: 1, HCT116 wild type 21% O_2_; 2, HCT116 *hif1α^−/−^* 21% O_2_; 3, HCT116 wild type 1% O_2_; 4, HCT116 *hif1α^−/−^* 1% O_2_. NS, not significant.

Metabolites	Source of Variation (*p*-value)			Bonferroni multiple comparisons Post-test analysis
	HIF1-α	O_2_ tension	Interaction	
Acetate	NS	NS	NS	
Omega-3	<0.05	<0.05	NS	1-2: <0.05; 1-3: <0.05; 2-4: 0.01; 3-4: <0.05
Total Fatty acids	<0.05	<0.01	NS	1-2: <0.01; 1-3: <0.05; 2-4: 0.01; 3-4: <0.001
MUFAs	<0.0001	<0.01	NS	1-2: <0.001; 1-3:<0.05; 2-4: <0.01; 3-4: <0.001
DiUFAs	<0.01	<0.01	NS	1-2: <0.01; 1-3:<0.05; 2-4: <0.05; 3-4: <0.01
PUFAs	<0.01	<0.01	NS	1-2: <0.01; 1-3:<0.05; 2-4: <0.05; 3-4: <0.01
Palmitate	NS	<0.01	NS	1-2: <0.05; 1-3: <0.01; 2-4: <0.01
Stearate	NS	<0.01	NS	1-2: <0.05; 1-3: <0.01; 2-4: <0.01
Oleate	<0.05	<0.05	NS	1-2: <0.05; 1-3: <0.05; 2-4: <0.01; 3-4: <0.05
Stearate/Palmitate	NS	<0.01	NS	1-3: <0.05; 2-4: <0.01
Oleate/Stearate	<0.01	NS	NS	1-3: <0.05; 3-4: <0.05
TAG	<0.0001	<0.001	NS	1-2: <0.01; 1-3: <0.05; 2-4: <0.01; 3-4: <0.01
Glycerol	<0.01	NS	NS	1-3: <0.05
Glycerophosphate	<0.05	NS	NS	1-3: =0.05
Choline	<0.001	<0.001	<0.05	2-4: <0.001; 3-4: <0.001;
Phosphocholine	<0.001	<0.01	<0.05	2-4: <0.01; 3-4: <0.01
Phosphatidylcholine	<0.001	<0.001	<0.05	2-4: <0.01; 3-4: <0.01
MAG	<0.05	<0.01	0.05	2-4: <0.05; 3-4: <0.05
PAF C16	NS	<0.05	NS	1-3: <0.05; 2-4: <0.01

**Table 2:** Analysis of lipid processing enzymes intensities performed by two way ANOVA Statistical significance is shown for the source of variation (HIF1-α, O_2_ tension or their interaction); the statistical significance within the groups is shown after Bonferroni multiple comparisons post-test analysis. Abbreviations: 1, HCT116 wild type 21% O_2_; 2, HCT116 hif1α^−/−^ 21% O_2_; 3, HCT116 wild type 1% O_2_; 4, HCT116 hif1α^−/−^ 1% O_2_. NS, not significant.

Enzyme	Source of Variation (*p-*value)			Bonferroni multiple comparisons Post-test analysis
	HIF1-α	O_2_ tension	Interaction	
Acetyl-CoA Carboxylase	<0.0001	0.001	NS	1-2: <0.001; 1-3: <0.001; 2-4: <0.001; 3-4: <0.01
Acetyl-CoA actyltransferase	<0.0001	NS	NS	1-2: <0.001; 3-4: <0.001
Stearoyl-CoA desaturase	NS	<0.0001	NS	1-3: <0.01; 2-4: <0.001; 3-4: <0.01
Phospholipase D3	NS	<0.001	NS	1-3: <0.05; 2-4: <0.001

### Hypoxic profile of fatty acids

**Figure 4:**
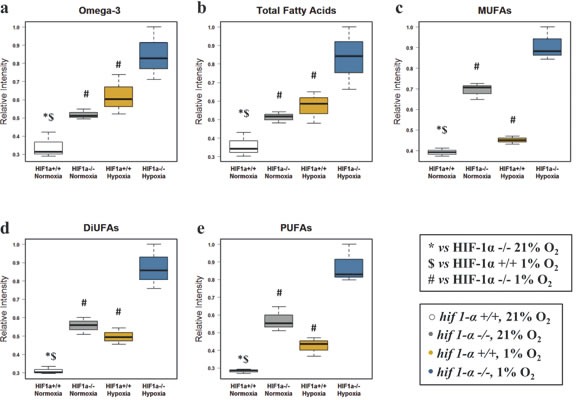
Fatty acid profile of HCT116 colorectal cancer cells in hypoxia (a) Omega-3 fatty acids (b) total fatty acids (c) MUFAs (d) DiUFAs and (e) PUFAs were detected by ^1^H-NMR in the organic phase. Normalized relative intensities are reported as mean ±sd (n=3).

**Figure 5:**
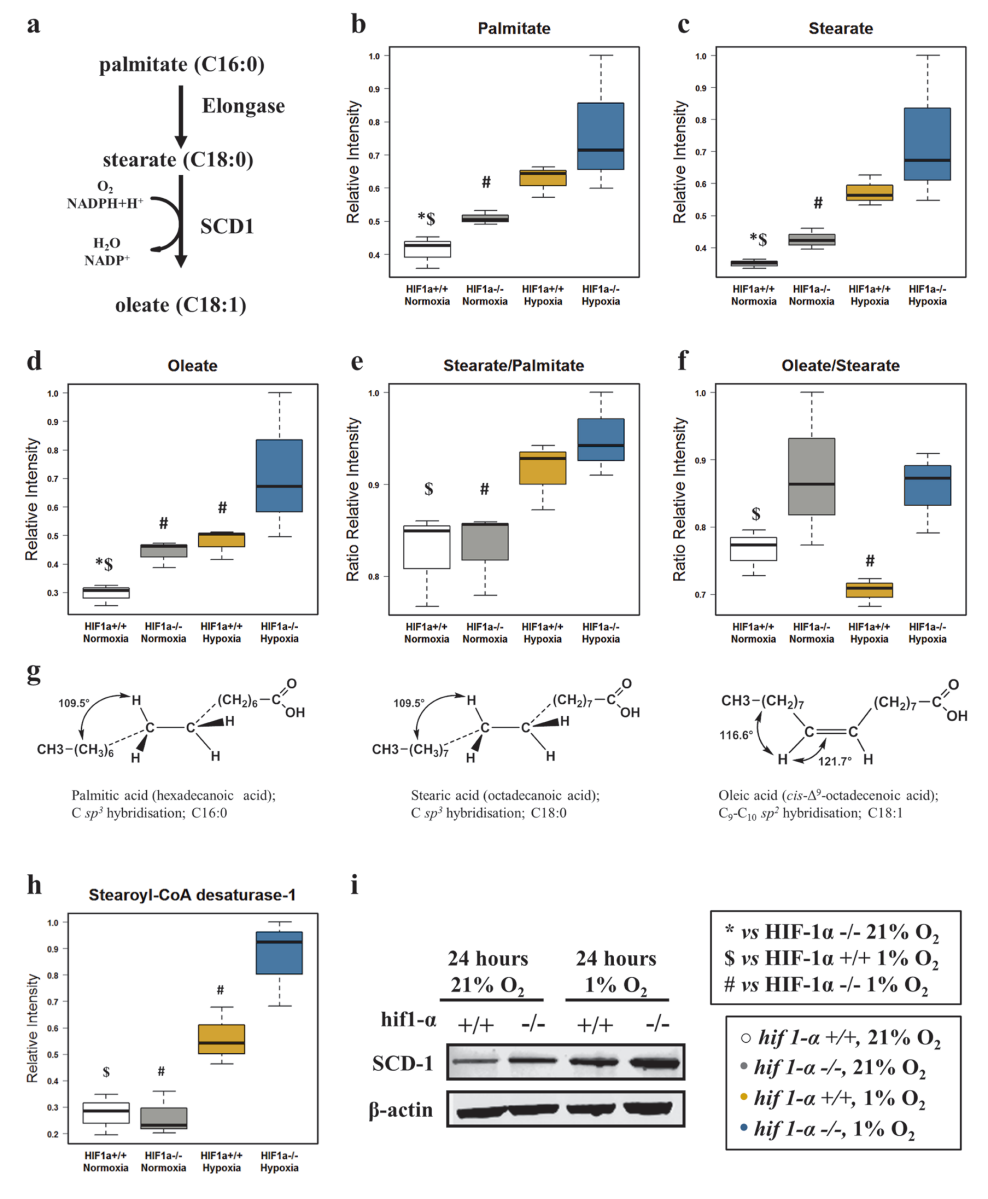
Fatty acid elongation and desaturation in HCT116 colorectal cancer cells in hypoxia (a) Palmitate is the first fatty acid formed by the activity of FASN after 7 cycles of condensing molecules of acetyl-CoA. This can undergo further elongation and/or desaturation to form the intracellular pool of fatty acids. SCD-1, by adding a double bond to stearate in a reaction requiring O_2_ and NADPH+H^+^, forms oleate. (b and c) HIF1α independent distribution of normalized relative intensities levels of saturated fatty acids palmitate (C:16) and stearate (C:18) detected by GC/MS-TOF and reported as mean ±sd (n=3). (d) Normalized relative intensities of HIF1α dependent unsaturated fatty acid oleate detected by GC/MS-TOF reported as mean ±sd (n=3). (e) Stearate/Palmitate ratio calculated on the normalized relative intensities and reported as mean ±sd showing that hypoxia favors the elongation of palmitate (n=3). (f) Oleate/Stearate ratio is known as desaturation index (DI) and is a parameter used to assess SCD-1 activity. Levels are calculated on the normalized relative intensities and reported as mean ±sd (n=3). (g) Tridimensional tetrameric saturated molecular structures of palmitate and stearate showing the hybridization *sp3*. Oleate *cis* planar *sp2* hybridized structure presenting an unsaturated double bond in position C_9_-C_10_. (h and i) SCD-1, levels detected by label-free quantitative proteomics analysis (h) and validated by western blot analysis (i) in HCT116 cells (n=3). Normalized proteomics intensities are reported as mean ±sd (n=3). A representative immuno blot for SCD1 using the M38 antibody is shown in (i). Similar results were observed using the R347 antibody (data not shown).

Due to their physico-chemical characteristics, FAs are fundamental constituents of complex lipids (Figure 3a). Under hypoxic conditions, total FAs, omega-3 FAs, mono, di and poly-unsaturated FAs (MUFAs, DiUFAs and PUFAs) accumulated significantly (figures 4 and 5). Their marked accumulation, observed in *hif1α^−/−^* hypoxic cells, demonstrated that HIF1α suppressed their levels. The same pattern was observed for their basal levels in normoxia (Figure 4a, b, c, d and e; Table 1). Palmitate and stearate accumulated in hypoxia in a HIF1α-independent manner. However, the presence of HIF1α in normoxic wild type cells decreased their levels. As observed for MUFAs, the unsaturated fatty acid oleate showed an accumulation in hypoxia, but HIF1α reduced oleate levels in hypoxic wild type HCT116 cells a distribution observed also in normoxia (Figure 5a, b, c, d and e; Table 2). We also examined the oleate/stearate ratio comparing intensities of unsaturated planar oleate *vs* the saturated tetramer stearate (Figure 5f and g), also known as the “desaturation index” (DI) [25]. The DI showed a HIF1α-dependent profile, decreasing significantly only in wild type hypoxic cells (Figure 5f; Table 1), indicating an altered stearoyl-CoA desaturase-1 (SCD-1) activity under these conditions. Hypoxia-dependent accumulation of this enzyme catalyzing oleate formation was more pronounced in *hif1α^−/−^* hypoxic cells (Figure 5h and i; Table 2). Thus, HIF1α suppresses SCD-1 levels in hypoxic wild type cells. No difference was found for basal SCD-1 levels in normoxia. SCD-1 levels were consistent between proteomics and western blot based assays (Figure 5g, h and i; Table 2).

### Hypoxic metabolism of fatty acid derivatives

**Figure 6:**
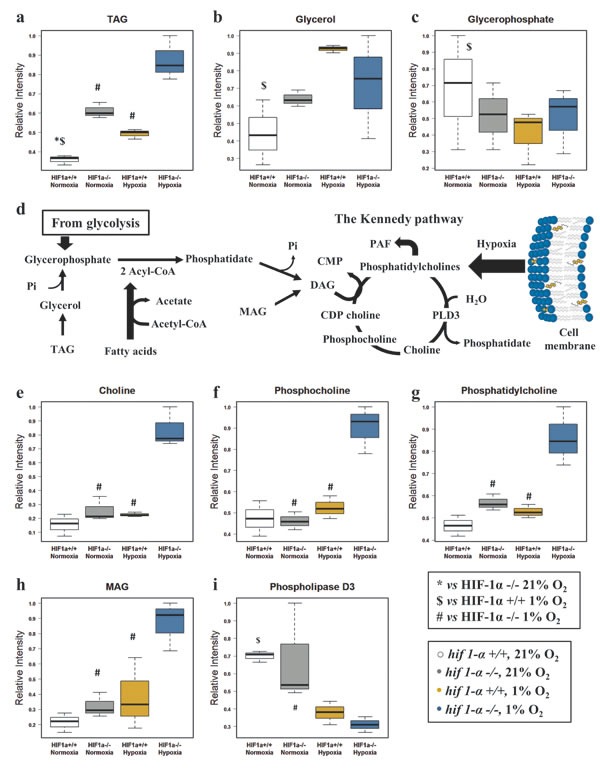
Glycerol derivatives and phospholipids are dependent on HIF1α (a) TAG normalized levels detected by ^1^H-NMR in the organic phase of the cell extracts, reported as mean ±sd (n=3). (b) Glycerol normalized levels detected by ^1^H-NMR in the aqueous phase of the cell extracts, reported as mean ±sd (n=3). (c) Glycerophosphate normalized levels detected by CE/MS in the aqueous phase of the cell extracts, reported as mean ±sd (n=3). (d) Metabolic pathway generating the precursors utilized in the Kennedy pathway. Abbreviations:CMP, Cytidine monophosphate; Pi, phosphate inorganic; CDP-choline, Cytidine-diphosphocholine; PLD3, Phospholipase D3. (e) Choline, (f) phosphocholine, (g) phosphatidylcholine and (h) MAG normalized levels reported as mean ±sd, were detected by ^1^H-NMR in the organic and aqueous phase of the cell extracts (n=3). (i) Phospholipase D3 normalized levels detected by label-free quantitative proteomics analysis in HCT116 cells data are reported as mean ±sd (n=3).

Hypoxia caused an increase of TAG levels and the absence of HIF1α strongly reinforced this effect in *hif1α^−/−^* cells. This effect was also observed in *hif1α^−/−^* normoxic cells, indicating that HIF1α suppresses hypoxic TAG accumulation (Figure 6a; Table 1). Hydrolysis of TAG generates free glycerol that can be phosphorylated to glycerophosphate. Interestingly, the level of these two metabolites showed an opposite distribution with HIF1α causing an accumulation of glycerol and a suppression of glycerophosphate in hypoxic wild type HCT116 cells (Figure 6b and c; Table 1). The levels of MAG, choline (Cho) and phopsphocholine (PCho), all involved in phosphatidylcholine (PC) biosynthesis through the Kennedy pathway (Figure 6d), were unaltered in normoxia and hypoxia-induced wild type cells. Surprisingly, only *hif1α^−/−^* cells accumulated MAG, Cho, PCho and PC levels under hypoxia, thus underlining the suppressive HIF1α-dependent effect on this metabolic pathway (Figure 6e, f, g and h; Table 1). The levels of phospholipase D3 (PLD3), mediating PC catabolism resulting in phosphatidate and Cho (Figure 6d), were down regulated in both wild type and *hif1α^−/−^* hypoxic cells in a HIF1α-independent manner. Levels were unchanged under normoxia (Figure 6i; Table 2).

### Platelet activation factor 16 (PAFC16) is regulated in hypoxia

**Figure 7:**
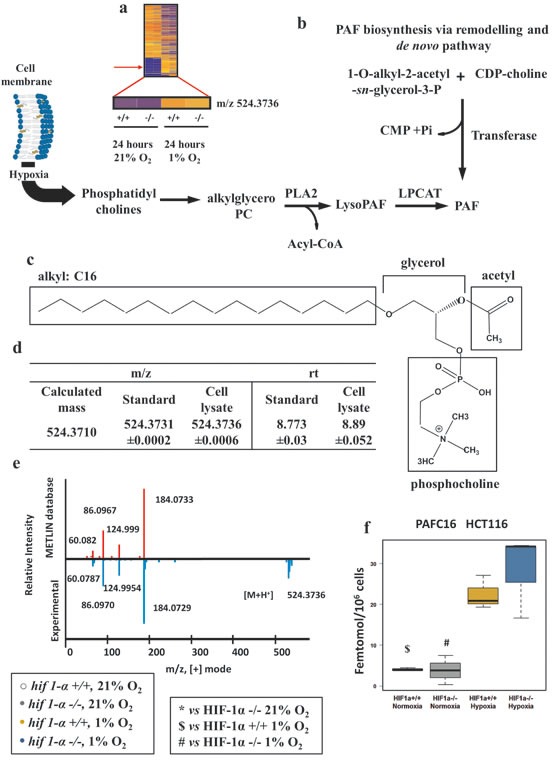
Platelet activating factor C16 (PAFC16) is regulated in hypoxia independently of HIF1α (a) Heat map of organic extract molecular features showing the detection of the *m/z*=524.3736 by nanoflow LC/MS positive mode. (b) PAF biosynthesis via *de novo* pathway and via remodeling pathways. Hypoxia, favoring cell membrane remodeling releases PC the substrate used for PAF biosynthesis. Abbreviations: LPCAT, acetyltransferase; PLA2, phospholipase A2; CMP, Cytidine monophosphate; Pi, phosphate inorganic; CDP-choline, Cytidine-diphosphocholine. (c) Molecular structure of PAFC16. In hypoxia PC provides the skeleton of PAFC16 (glycerol and phosphocholine); the characteristic saturated hexadecil moiety (16:0) is a derivative of palmitate reduction. Acetyl deriving from acetyl-CoA completes the structure of PAFC16. (d) PAFC16 identification was performed by LC/MS QTOF nanoflow using mass matching and retention time comparison. (e) Tandem mass (MS/MS) spectra performed by LC/MS QTOF nanoflow of experimental detection of *m/z*=524.3736 [M+H]^+^ and comparison matching with METLIN database was the third parameter used for PAFC16 identification. (f) Intracellular PAFC16 concentrations reported as femtomol/10^6^ cells data are shown as mean ±sd, intensities were quantified by LC/MS Q Exactive (n=3). Concentration was calculated interpolating a linear range standard curve with the unknown quantified relative intensities.

To extend the pool of identified molecular features with an altered profile in hypoxia (Figure 1d and 7a), we performed METLIN database searches, and observed an accurate mass match for m/z 524.3736 [M+H]^+^ corresponding to platelet activating factor 16 (PAFC16), a C16:0 monoalkylglycerol ether-derivative, esterified with an acetyl group in C_2_ and condensed with a Cho polar head in C_3_ (Figure 7b and c). PAF is a lipid synthesized through the *(i) de novo* pathway where a transferase adds PC to the *sn*-3 site of the 1-O-alkyl-2-acetyl-sn-glycerol-3-P and *(ii)* remodeling pathway where PC is converted to lyso-PAF through a phospholipase D mediated loss of an acyl group in *sn*-2 and subsequently re-acetylated (Figure 7b). PAFC16 identity was confirmed by matching the m/z observed in biological experiments with the calculated mass (Δppm <5) and by comparing PAFC16 LC retention times between biological experiments and standard. Finally, PAFC16 MS/MS spectra were matched between biological samples and the MS/MS spectrum reported in the METLIN data base, demonstrating identical profiles of fragmented ions (Figure 7d and e; table 3). Wild type and *hif1α^−/−^* hypoxic cells showed both a clear HIF1α-independent accumulation of this bioactive lipid (Figure 7f). PAFC18 and PAF catabolism products Lyso-PAFC16 and Lyso-PAFC18 did not show any significant difference in our experiments (data not shown). HIF1α-independent PAFC16 accumulation in hypoxia was confirmed in *hif1α^KD^* DLD-1 and SW1222 colorectal cancer cells (figure S1). Also, a HIF-independent accumulation was observed in *hif2α^KD^* and *hif1/2α^KD^* DLD-1 cell lines after 24 hours of hypoxia (figure S2). Normoxic basal levels of PAFC16 were in the range of 3.8 to 13.3 femtomol/10^6^ cells. Under hypoxia, levels increased to the range of 21.7 to 59 femtomol/10^6^ cells. No statistical differences were observed when we compared PAFC16 levels within the different parental cell lines (wild type, *hif1α^−/−^*, *hif1αKD*, *hif2α^KD^* and *hif1/2α^KD^*) in normoxia and in hypoxia (table S3).

**Table:** Multiplatform metabolomics Instrumental, method of detection, parameters used for the metabolites identification (RA: resonance assignment; EI: electro-impact spectrum; RI: retention index; AMRT: accurate mass retention time; MS/MS analysis) and matrix utilized for the detection of lipophilic metabolites.

Metabolite	Detection Method	Identification	Matrix
Acetate	^1^H-NMR	RA spectrum	Aqueous
Omega 3 FAs	^1^H-NMR	RA spectrum	Organic
Total Fatty acid	^1^H-NMR	RA spectrum	Organic
MUFAs	^1^H-NMR	RA spectrum	Organic
DiUFAs	^1^H-NMR	RA spectrum	Organic
PUFAs	^1^H-NMR	RA spectrum	Organic
Palmitate	GC/MS	EI spectrum, RI	Aqueous
Stearate	GC/MS	EI spectrum, RI	Aqueous
Oleate	GC/MS	EI spectrum, RI	Aqueous
Glycerol	^1^H-NMR	RA spectrum	Aqueous
Glycerophosphate	CE/MS	AMRT	Aqueous
MAG	^1^H-NMR	RA spectrum	Organic
TAG	^1^H-NMR	RA spectrum	Organic
Choline	^1^H-NMR	RA spectrum	Aqueous
Phosphocholine	^1^H-NMR	RA spectrum	Organic
Phosphatidylcholine	^1^H-NMR	RA spectrum	Organic
PAF C16	LC/MS	AMRT, MS/MS	Organic

**Figure 8:**
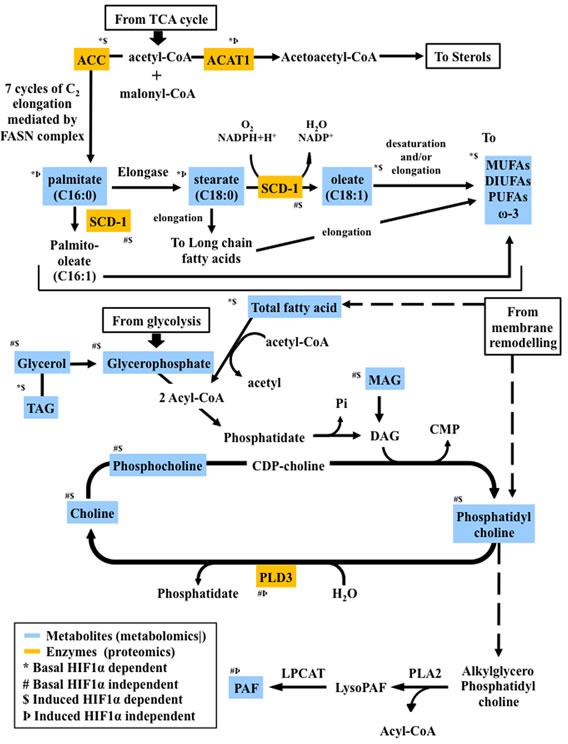
Proteo-metabolomic integrative overview of the altered metabolic pathways under hypoxia and their dependence on HIF1α

### Gene hypoxia signature and lipid metabolism

**Figure 9:**
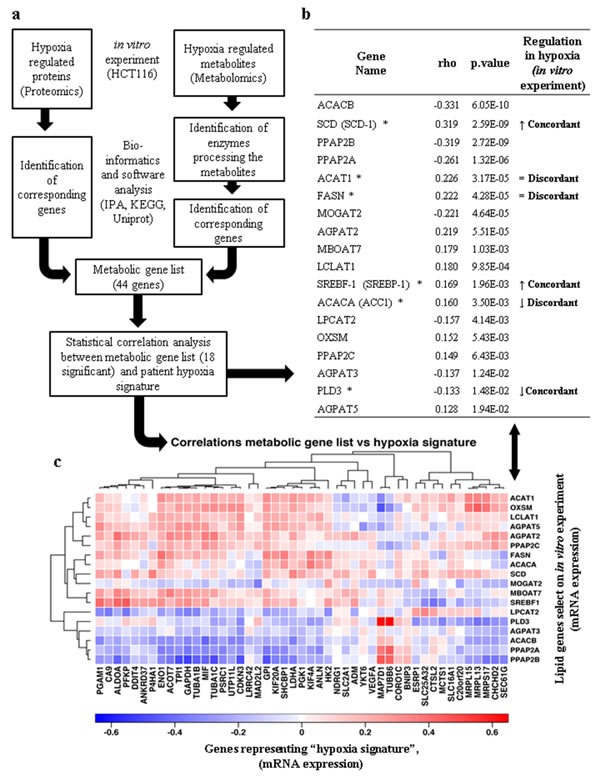
Translational analysis of hypoxia mediated lipid metabolism (a) Multi-omics biology workflow to select genes involved in lipid metabolism and hypoxia. (b) Relationship between the profiles of hypoxia regulated lipid metabolism genes in primary colon cancer [31] and genes selected from the proteomics/metabolomics experiments in this study (figure S4). Only the 18 genes for which the Spearman's ρ correlation coefficients were significant are shown (*p*-value <0.05). (↑) indicates protein positively regulated by hypoxia in HCT116 wild type cells. (↓) indicates protein negatively regulated by hypoxia in HCT116 wild type cells. (=) indicates no change in protein regulation in hypoxic HCT116 wild type cells. (*) Indicates proteins that have been verified for expression in HCT116 cells. The concordance or discordance of the two data sets is indicated. (c) Heatmap illustrating the correlative gene expression profile of lipid metabolism genes selected from cellular experiments (listed in (b)) compared to 47 genes representing a “hypoxia signature” based on 333 colorectal carcinoma patients previously analysed by TCGA [31].

To place our cellular experiments in a cancer related context, genes relevant to lipid metabolism observed in our data and described in public databases were selected and their levels of mRNA expression evaluated in a colorectal cancer patient cohort (Figure S4) [31]. The selection criteria for lipid associated genes were based on (i) hypoxia regulated proteins in HCT116 cells observed in our proteomics experiments; (ii) enzymes that process metabolites for which we observed altered levels in hypoxia in metabolomic experiments. A group of fourty-four genes fulfilled these criteria (Figures 9a and S4a). Spearman's ρ analysis allowed the assessment of the correlation between the mRNA expression of the forty-four selected lipid metabolism related genes with the mRNA levels of a “hypoxia signature” defining genes observed in the patient cohort (S4b, c and d). Eighteen out of the forty-four genes showing a *p*-value that is statistically significant are reported in figure 9b and c. These were selected to be compared to the results observed in our experiments. The protein levels of SREBP-1, SCD-1, and PLD3 observed in HCT116 hypoxic cells (figures 3e, 5i and S6) correlated with the trend of mRNA expression related to the hypoxia signature. Interestingly, ACAT1, FASN and ACC1 (enzymes directly involved in acetyl-CoA metabolism) showed a discordant correlation between the protein and mRNA levels (figures 3c, d, e and 9b), suggesting these as possible key points for metabolic alteration in hypoxia. For the other twelve genes we did not observe any clear trend, suggesting that the levels of downstream metabolites might be determined by a complex synergy of enzyme regulation by posttranslational modifications and the interplay between catabolic and anabolic processes.

## DISCUSSION

Hypoxia is a hallmark of many human cancers, a consequence of cancer cell proliferation consuming oxygen and aberrant blood vessel development, leading to the local induction of the transcription factors HIF1α and HIF2α [28]. HIF1α regulates hundreds of genes, and many of them play a role in cancer metabolism [29]. O_2_-independent mechanisms can also stabilize HIF1α, i.e. mutations in the *Von Hippel-Lindau* (pVHL) tumor suppressor gene in renal carcinoma [32, 33]. Hypoxic adaptations of metabolic pathways such as glycolysis and the TCA cycle have been extensively described [1-6, 8, 28]. However, less is known about HIF-dependent pathways in regulating lipid metabolism in hypoxia. In our study, we found that the lipid profile of cancer cells exposed to hypoxic conditions undergoes profound changes following four major patterns as summarized in figures 2 and 8.

### HIF1α modulates the metabolic steps supplying acetyl-CoA for the *de novo* FAs biosynthesis

Acetyl-CoA, physiologically formed either by the citric acid cycle or by of FAs β oxidation, is the starting substrate for the synthesis of more complex molecules. In hypoxia, the reductive carboxylation pathway was recently shown to reduce glutamine to citrate and providing the predominant pathway for FAs production [34-37]. A hypoxic lipogenic phenotype was demonstrated to be the result of increased lipid scavenging activity in MDA-MB-468, HeLa and A549 cell lines, rather than an augmented lipogenesis [10, 25, 38]. This process requires transmembrane transporters such as the ABC superfamily (1, 2 and 8) that increase the intracellular lipid pool to support enhanced metabolic processes [39]. Consistent with this, we observed HIF1α-dependent reduction of ACC1 levels, which could limit the initial step in FAs biosynthesis, supporting the idea that cancer cells scavenge lipids from the extracellular environment [25, 38]. No differences were observed in acetate levels, suggesting a prompt utilization of acetyl-CoA either through the *de novo* FAs biosynthesis or through the sterol metabolism response. ACAT1, redirecting acetyl-CoA to sterol biosynthesis was accumulated to the same extent in wild type compared to *hif1α* ^−/−^ cells, a process not affected by hypoxia. as previously shown in human monocyte-derived macrophages [40, 41]. We observed that SREBP-1, an important regulator of lipogenesis and sterol response, is upregulated in hypoxia as reported previously [23, 42]. Together, our data shows that HIF1α suppresses the metabolic steps supplying substrates for FAs biosynthesis.

### Role of HIF1α in the *de novo* biosynthesis of FAs

Hypoxia has been suggested to trigger FASN activation that depends on SREBP-1 through a process linked to HIF1α, PI3K-Akt-mTOR and Ras activation [2, 23, 43, 44]. In contrast to their normal counterparts, cancer cells rely on *de novo* FAs biosynthesis [7, 45]. mRNA levels of FASN, the key enzyme in this process, were induced in breast cancer lines in response to 48 h of hypoxia [20], while FASN expression was observed to be reduced after 12 h hypoxia in HepG2 cells [46]. However, we found no change in FASN protein expression after 24 h hypoxia, but we observed a hypoxia-induced FAs profile consistent with previously reported data showing lymphoma cells scavenging fatty acids in hypoxia [25, 47]. Depletion of enzymes involved in FAs metabolism, including ACC1, FASN and SCD-1, augmented cytotoxicity in HCT116 cells due to an increase of basal apoptosis, which could be reversed by addition of exogenous FAs [40, 41, 48].

Saturated FAs can be metabolized to MUFAs by SCD-1, a key regulator of this process. SCD-1 is an O_2_-dependent enzyme specific for palmitate and stearate, adding a double bond nearly always in “*cis*”- Δ9 and thereby forming palmitoleate or oleate, respectively [48-50]. SCD-1 was found to be constitutively expressed in several human cancers [48, 51, 52]. MUFAs accumulation in cancer cells was shown to be implicated in carcinogenesis in animal models, but on the other hand, a lower SCD-1 expression/activity may reduce risk of breast cancer [40, 48, 53].

We observed an accumulation of SCD-1 in wild type HCT116 cells under hypoxia, which was further enhanced in the absence of HIF1α. DI (oleate/stearate ratio a parameter used as a surrogate for SCD-1 activity) showed a comparable distribution in normoxic and hypoxic *hif1α^−/−^* HCT116 cells, indicating that stearate and oleate levels were regulated similarly under these conditions (figure 5). In contrast, hypoxic wild type cells showed a reduction of the DI determined by the limited conversion of stearate to oleate in hypoxia, consistent with previously reported results demonstrating reduced SCD-1 enzymatic activity when oxygen levels are low [25, 48]. This is compensated by the increase of SCD-1 levels in absence of HIF1α under hypoxia. Taken together, we conclude that both SCD-1 activity and expression levels are manly controlled by HIF1α.

### Phosphoglycerolipid metabolism in hypoxia

Phospholipids are the main constituents of cell membranes, and the degree of FAs desaturation affects their biophysical properties, which in turn influences many membrane-associated functions. Hypoxia induces membrane remodeling, a process favoring phospholipid release, in particular PC [54]. HIF1α mediates the induction of lipin which catalyzes the conversion of phosphatidic acid to diacylglycerol, thereby contributing to TAG accumulation. HIF1α also controls PPRγ, a nuclear receptor controlling acyl-CoA oxidase expression levels, which in turn provides glycerophosphate for FAs and PCho condensation [11, 55, 56]. We observed a HIF1α-dependent downregulation of hypoxia-induced glycerophosphate levels, concordant with HIF1α being a primary regulator of glycolytic processes [57]. In addition, our results showing unchanged levels of PCho in hypoxic wild type HCT116 cells, are in line with previous studies that observed no increase of cellular uptake of Cho in hypoxia [58]. PCho has been suggested as a potential biomarker for breast cancer reflecting an upregulation of specific Cho transporters [59]. Hypoxia-induced membrane remodeling releases PC, and high levels of PC containing unsaturated FAs as well as the enzyme SCD-1 were shown to be localized in cancerous areas of human breast carcinoma tissue rather than the stromal areas. Also, stearate levels were significantly lower in membrane phospholipid mixtures extracted from breast carcinoma tissues obtained at the time of surgery in patients who developed metastasis [60, 61].

### PAF pathway regulation in hypoxia independently of HIF1α or HIF2α

Intracellular availability of PC can provide substrates for PAF biosynthesis through the remodeling pathway: a primary source for PAF under pathological conditions also activated by inflammatory agents [62]. PAF was originally described as a platelet aggregation inducer, but it is also involved in many other processes such as cell proliferation, migration, neoangiogenesis, inflammation and cancer. The biological responses, induced at sub-nM concentrations, occur through the stimulation of a G-protein-coupled PAF receptor upstream of second messengers (cAMP, IP_3_ DAG), protein kinases (MAPK, PKC) and tyrosine kinase (PLCγ and PI3K) [62, 63]. We found intracellular PAFC16 levels in the range of 3.8 to 59 femtomol/10^6^ cells equivalent to 1.9 to 30.9 pg/10^6^ cells, similar to previous observations [64]. HIF1α and HIF2α have been suggested to play different roles in lipid metabolism. Inactivation of HIF2α alters hepatic lipid metabolism and FAs levels in mice [65]. Our study shows that PAFC16 accumulation is both HIF1α and HIF2α independent in hypoxia, an effect observed in multiple cell lines. A role for PAF in oncogenic transformation, metastasis and angiogenesis was suggested in many types of tumors including ovarian, breast, colorectal carcinoma and prostate cancer [66-70]. Additionally, PAF levels were found to be higher in liver metastasis as compared to primary colorectal tumor of the same patient's cohort [67]. The characteristic saturated hexadecil moiety is important for PAF activity, and a C18:0 derivative is also active [62]. Intracellular PAF is regulated by the balance of anabolic and catabolic mechanisms. Catabolism, mediated by acetyl hydrolase, forms lyso-PAF and acetate [62, 71]. In our experiments, no changes in hypoxia were observed for lyso-PAFC16 and acetate. Phospholipase D transforms lyso-PAF to choline and phosphatidic acid [62, 72], and our data showed a clear reduction of PLD3 protein levels in hypoxia, a trend observed also for PLD3 mRNA *vs* hypoxia signature in colorectal cancer patients [73]. Finally, the alkylglycerol specific monooxygenase (MO) cleaving the *O*-alkyl moiety of lyso-PAF is an enzyme requiring O_2_ [71]. Our data suggest that the HIF-independent hypoxic accumulation of PAFC16 in colorectal cancer cells could be due to a reduction of PAFC16 catabolism as a result of decrease in this enzyme activity. A decrease in MO, including heme-dependent MO, flavin-dependent MO, copper-dependent MO, non-heme/iron-dependent MO and pterin-dependent MO can be active at other steps of lipid metabolism and could also contribute.

In summary, our data sheds new light on a lipogenic phenotype that is induced in cancer cells under hypoxic conditions. Many biochemical processes involved in lipid metabolism are modulated under these conditions, such as *de novo* FAs biosynthesis, desaturation processes and phospho-derivatives biosynthesis, resulting in a complex alteration of the downstream levels of lipids. Our data show a key effect of hypoxia induced but HIF independent pathways, potentially through changes in mono oxygenase enzyme activity. Our findings show the prominent role of hypoxia in lipid metabolism, and provide the framework for alternative therapeutic strategies based on the interference with cancer-specific metabolic adaptation pathways.

## MATERIALS AND METHODS

### Cell culture and cell cycle analysis

HCT116 wild type cell lines were obtained from Cancer Research UK Cell Services while HCT116 *hif1α^−/−^* cells were kindly provided [30] and SW1222 and DLD1 were kindly provided by Professor Walter Bodmer's laboratory. Cells were grown in Dulbecco's modified Eagle's medium with low glucose (4 mM) DMEM (GIBCO-BRL) supplemented with L-glutamine 2mM from (Sigma, UK), 10% fetal bovine serum, penicillin / streptomycin (10 U/ml and 10 mg/mL, respectively) (Sigma, UK). HCT116, SW1222 and DLD1 wild type, *hif1α^−/−^*, *hif1α^KD^*, *hif2α^KD^* and *hif1/2α^KD^* cells were plated in 100-mm dishes allowing adherence and one doubling cycle; cell lines were further maintained under normoxic conditions for 24 hours or exposed for 24 hours to hypoxia (1% oxygen) using a hypoxic workstation (Ruskinn Technologies) and collected at 80% of confluence. After cells were harvested counted and fixed by rapid submersion in ice-cold 85% ethanol, DNA was stained with 0.25 mg/ml propidium iodide, 0.05 mg/ml RNase, 0.1% Triton X-100 in citrate buffer, pH 7.8, and analyzed on a Becton Dickinson FACScan (Becton Dickinson, San Jose, CA, USA).

### siRNA transfection

Colorectal carcinoma cell lines SW1222 and DLD1, 1-2×10^6^ cells were reverse transfected with 20nM siRNA using RNA Lipofectamine RNAimax (Invitrogen) in 10 cm plates, following the manufacturer's protocol. Cells were transfected with control siRNA, Hif1α siRNA and Hif2α siRNA. Forty eight hours post transfection, cells were incubated either in normoxic or hypoxic conditions for 24hrs. Cells numbers were counted and collected for RNA and Lipid extraction. The siRNA sequence are as follows: control 5′- AUGACGACCUGCGUGUCGU-3′; pool of three *HIF1α siRNAs* 5′- caagcaactgtcatatata-3′, 5′-tgccaccactgatgaatta-3′ and 5′-tgactccagctattcaccaa-3′; pool of three *HIF2α siRNAs* 5′-TAACGACCTGAAGATTGAA-3′, 5′-CAAGCCACTGAGCGCAAAT-3′ and 5′-TGAATTCTACCATGCGCTA-3′.

### Immunoblotting analysis

Wild type, *hif1α^−/−^*, *hif1α^KD,^ hif2α^KD^* and *hif1/2α^KD^* (HCT116, LDL-1 and SW1222) cells were washed with cold PBS and lysed with RIPA buffer (25mM Tris-HCl (pH 7.6), 150mM NaCl, 1% NP-40, 1% sodium deoxycholate, 0.1% SDS) containing protease (Roche, USA) and phosphatase inhibitor cocktail (Sigma, UK). Protein concentrations were quantified using a BCA protein assay kit (Thermo Fisher Scientific, Cramlington, UK). Samples containing 20μg of protein were in Laemmli buffer and separated using 8-12% pre-cast SDS-PAGE gels (Bio-Rad, USA). Proteins were transferred to PVDF membranes (Millipore, USA), blocked for 1 hour in transfer buffer (25 mM Tris, pH 7.5, 0.15 M NaCl, 0.05% Tween 20) 5% milk. Membranes were incubated overnight at 4°C with mouse monoclonal anti-Human HIF-1α (BD Biosciences, UK), rabbit anti-Human HIF-2α (Novus, Biologicals, Cambridge, UK), rabbit anti-Human ACC1, FASN, SREBP-1 and SCD-1 (M38 and R347) (Cell Signaling Technology, UK). Membranes were blotted with HRP-conjugated secondary antibody (Dako, UK) and washed for an additional hour. Blots were developed on Kodak film (Sigma, Steinheim, Germany) using ECL plus reagent (GE Healthcare, UK). Image J software was used to quantify HIF 1 α and 2α band intensities after beta-actin normalization.

### Sample preparation for metabolomics assessment

Sample treatment was carried out in the corresponding cell experimental conditions (normoxia and hypoxia oxygen 1%) with working solutions previously equilibrated to the oxygen levels when needed. Cells were washed with cold PBS, harvested and resuspended. An aliquot for each biological replicate was utilized for cell counting. Cells were collected, pelleted for 5 min at 1,500g and the media aspirated. Cells were washed with ice cold PBS and metabolites extracted by adding a sequence of CH_3_OH, Milli-Q H_2_O and CHCl_3_ (2:2:1) followed by mixing vigorously after the addition of each reagent and finally centrifuging at 2×10^5^ g for 15 minutes at 4 °C. The matrix obtained was formed by an aqueous and an organic layer separated by the proteins layer. Aqueous and organic layers were harvested for metabolomics analysis. Experiments were performed as three or five biological replicates and analyzed as three technical triplicates.

### Multiplatform metabolomics analysis

For the measurement of the saponified FAs, FAs-derivatives and other lipid molecules in cellular aqueous and organic extracts, a combination of different instrumental settings were used to increase the coverage of a cell's metabolome and to expand the analysis to a larger range of classes of metabolites with different physico-chemical characteristics as summarized in table 3.

#### ^1^H-NMR metabolomics analysis

For ^1^H-NMR measurement the lyophilized hydrophilic and lipid fractions were prepared and analyzed as previously described [74]. In brief, the aqueous extract was reconstituted in phosphate buffer in D_2_O containing trisilylpropionic acid while the organic phase lyophilized was resuspended in a solution CDCl_3_/CD_3_OD (2:1) containing tetramethylsilane and transferred into 5-mm NMR glass tubes for the analysis performed by a Bruker Avance III-600 spectrometer equipped with an inverse TCI 5 mm cryoprobe.

#### LC/MS nano-flow and CE/MS metabolomics analysis

Aqueous fractions were collected as described above and transferred to an ultrafiltration tube and the solution filtered by centrifugation at 9,000 g for 2 hours at 4 °C. 350 μl were recovered after filtration and concentrated during 3 hours and further resuspended in 50 μl of Milli-Q H_2_O for the LC/MS and CE/MS analysis. Organic fractions were concentrated and resuspended in isopropanol 70% (Sigma, UK), and the mixture were analyzed by nanoflow LC/MS. LC/MS was performed by liquid chromatography coupled to a quadrupole time of-flight mass spectrometer (Agilent Chip/6250 QTOF-MS) using a Zorbax 80 SB-C18 Chip Agilent (5μm 150mm x 75μm 2.5mm, 500nl) using a gradient of buffers A (2% acetonitrile, Millipore, USA, 98% Milli-Q H_2_O and 0.1% Formic Acid Sigma, UK) and B (95% acetonitrile, 5% Milli-Q H_2_O and 0.1% Formic Acid Sigma) ; B: 0% to 40%, minute 0 to 11; 40% to 100%, minute 11 to 14; 100% minute 14 to 15 and 100% to 0%, minute 14 to 15. The CE/MS analysis was performed using an Agilent G1603A CE-TOFMS system as previously described [75, 76].

#### GC/MS-TOF metabolomics analysis

Aqueous fraction was collected, lyophilized and incubated for 1.5 h at 37 °C with 50 μl of a mixture of 40 mg/mL of Methoxyamine hydrochloride 98% (Aldrich) and N-Methyl-N (trimethylsilyl) trifluoroacetamide (synthesis grade, Aldrich) *in p*yridine ACS reagent, >99% (Sigma, UK) followed by 30 μl of FAMES 40 ppm in TMS (Sigma, UK). The solution was shacked for 10 minutes at 37 °C and incubated in for 1 hour in a dark environment. Samples were centrifuged and 1 μl was used for the analysis. Untargeted, quantitative analysis was performed using a GC-TOF MS Pegasus 4D system (Leco Instruments, St. Joseph, MI, US) supported by a capillary column DB-5MS-DG (30m length x 0.25 mm DI, 0.25 μm film thickness, Agilent Technologies, Santa Clara, US). GC conditions: 1μl was injected at a constant carrier gas (helium 99.9995% purity) flow of 1ml/min. Inlet temperature of 250 °C, oven temperature, initially held at 50°C for 1min, then raised at 20°C/min to 330°C, and held for 5min. Total run time was of 20 min with a transfer line temperature of 250°C. MS conditions: ionization was performed by molecules electronic impact using a source temperature of 250°C, delay time of 330 sec, acquisition rate of 10 spec/sec, acquisition range from 85 to 500 m/z and voltage of 1600V.

#### Metabolites identification and quantification

Metabolites identification and quantification was performed as shown in Table 3 and raw intensities are reported in table S5. Resonance assignments (RA) was done on the basis of literature values and different database search engines from Bruker®, Chenomx Inc and in house lab libraries. Neat regions were selected for spectra integration and metabolites quantification as previously described [74]. Identification and quantification of deconvoluted GC/MS-TOF detected compounds was done by Bin Database [77], in basis to the EI spectrum and retention index (RI). CE-MSTOF data were processed by in house software based on the characteristic *m/z* matching and migration time with standard compounds. Peaks were exported for quantification as reported previously [75, 76]. LC/MS QTOF nanoflow based metabolite discovery and identification was performed as described in figure 6 using the PAFC16 pure compound (Cayman chemicals, USA).

#### Intracellular quantification of PAFC16

Organic fractions, processed as described before, were re-suspended in isopropanol 70% and analysed by LC/MS using a Dionex U3000 coupled directly to a Q-Exactive (Thermo) mass spectrometer system. Chromatography was performed using a Water Acquity UPLC BEH C18 1mm x 100mm reverse phase column with a 25min gradient. Three mobile phases were used: A (Milli-Q H_2_O with 0.1% Formic Acid Sigma, UK), B (acetonitrile with 0.1% Formic Acid Sigma) and C (50% Ethylacetate in acetonitrile, Sigma).

B: 0% to 25%, minute 0 to 5; 25% to 100%, minute 5 to 16; 100% minute 16 to 25; C: 0% to 100%, minute 16 to 25. PAFC16 quantification was calculated using a standard calibration curve obtained using an authentic standard at the following concentrations: 0, 1, 2.5, 5, 10, 25, 50, 75 and 100 nM. The linear regression equation was *Y* = 4E+06x + 8E+06 and calculated an r^2^=0.995.

### Proteomics analysis

Total cell lysates were reduced with DTT and cysteines alkylated with iodacetamide followed by precipitation and digestion with Trypsin (Promega) as described previously [78]. Resulting peptides were desalted (SOLA RP) before analysis. Mass spectrometric analysis of digested cell lysates was conducted on a Q-Exactive (Thermo) mass spectrometer with a resolution of 70,000 (at 200 M/z) coupled to a Dionex Ultimate 3000 UHPLC (Thermo) system. Peptide separation was archived on an Easy column (2μm Pepmap, C18, 75μm x 500mm) using a linear gradient from 2-40% of buffer B (composition as above) in 57 minutes. Precursors with an M/z between 380 and 1800 were selected for MS/MS with an isolation width of 1.6 Da and 28% normalized collision energy using the 15 most abundant precursor ions. Selected precursors (Threshold 10,000 counts) were excluded for 27s. MS/MS spectra were searched using the Mascot search engine (Matrixscience), and quantitation was performed using LC Progenesis software (Non-Linear Dynamics) as described [78]. Raw intensities are reported in table S6.

### Genomic analysis

Genome-scale analysis was conducted on “The Cancer Genome Atlas” (TCGA) cohort [73] a database based on the analysis of exome sequence, DNA copy number, promoter methylation, mRNA and microRNA expression characterize somatic alterations in colorectal carcinoma of 333 patients (data release 2014-01-15). Only 304 patients with known clinical outcome were included (figure S4). A Spearman's ρ correlation test was performed between the mRNA expression of 44 selected lipid metabolism and a published hypoxia signature consisting of 48 genes [31]. In this process for each patient a signature score was calculated by the median mRNA expression of the hypoxia genes. Bonferroni correction for multiple testing was applied to these correlations and only metabolic genes with p < 0.05 after correction were considered to have a significant correlation with the signature.

### Statistical analysis

MS and NMR derived normalized data are presented as mean (±standard deviation), with *p*-value less than 0.05 indicating statistical signiﬁcance, using R package. Significance of the two independent variables (HIF1α knockout and hypoxia) was evaluated by two-way analysis of variance. Effects of HIF and hypoxia were evaluated also as interaction of factor one (HIF1α knock out) and factor two (hypoxia). Bonferroni post-test multiple comparison analysis was applied to evaluate significance within groups as reported in Tables 1 and 2.

## SUPPLEMENTARY MATERIAL FIGURES AND TABLES


