# Supplementary material for: Hypoxia induces a lipogenic cancer cell phenotype via HIF1α-dependent and -independent pathways
Source: Oncotarget. 2014 Dec 11;6(4):1920–41. doi: 10.18632/oncotarget.3058 (PMC4385826; doi:10.18632/oncotarget.3058)
Supplement: Supplementary file 1 [file oncotarget-06-1920-s001.pdf]

Hypoxia induces a lipogenic cancer cell phenotype via HIF1α-dependent and -independent pathways

Supplementary Material

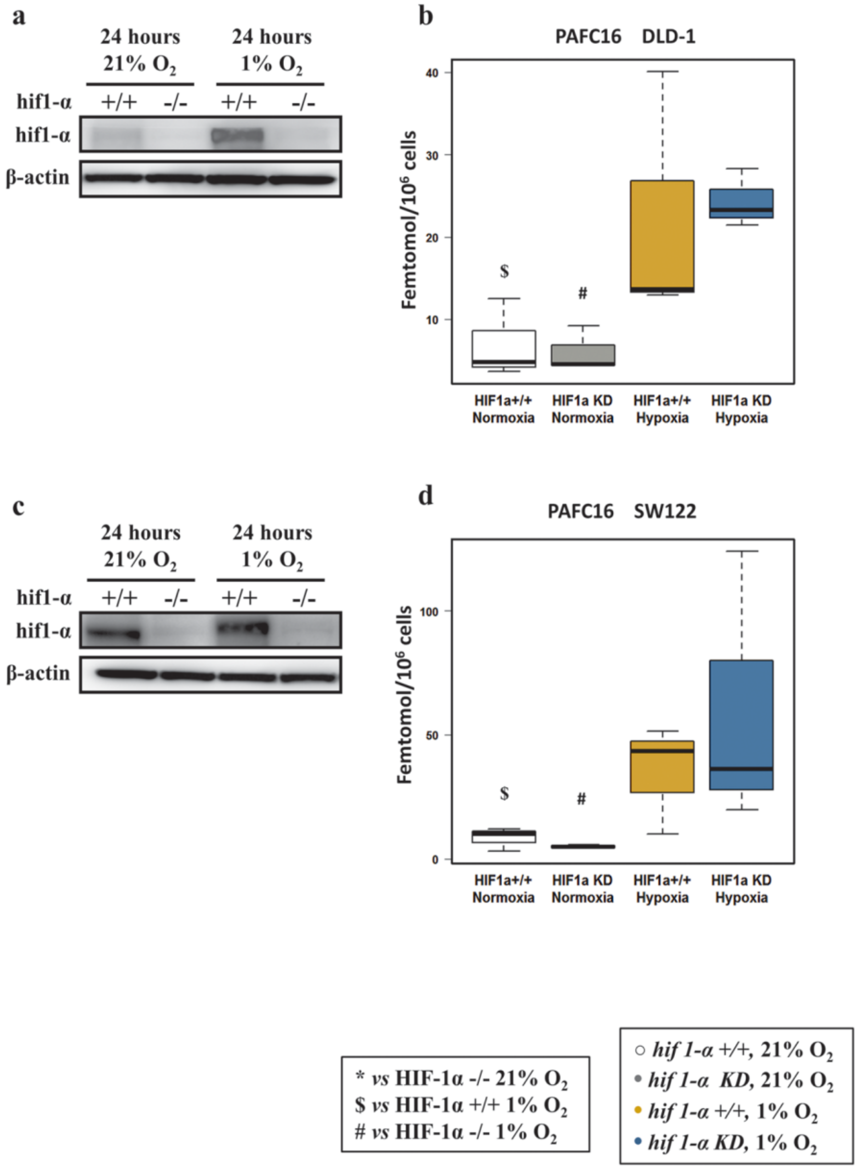

Supplemental S1: PAFC16 regulation in hypoxia is HIF1α independent: validation.

(a) DLD-1 colon cancer cells were treated as described. HIF1α levels, detected by western blot analysis, showed a significant knock down by specific siRNA and hypoxic induction in wild type

cells (n=3). **(b)** DLD-1 intracellular PAFC16 concentrations reported as femtomol/10^6 cells data are shown as mean ±sd, intensities were quantified by LC/MS Q exactive (n=3). Concentration was calculated interpolating a linear range standard curve with the unknown quantified relative intensities**.** **(c)** SW1222 colon cancer cells were treated as described in the text, HIF1α levels, , showed a significant knock down by specific siRNA and hypoxic induction in wild type cells (n=3). **(d)** SW1222 intracellular PAFC16 concentration reported as femtomol/10^6 cells data are shown as mean ±sd, intensities were quantified by LC/MS Q exactive (n=3).

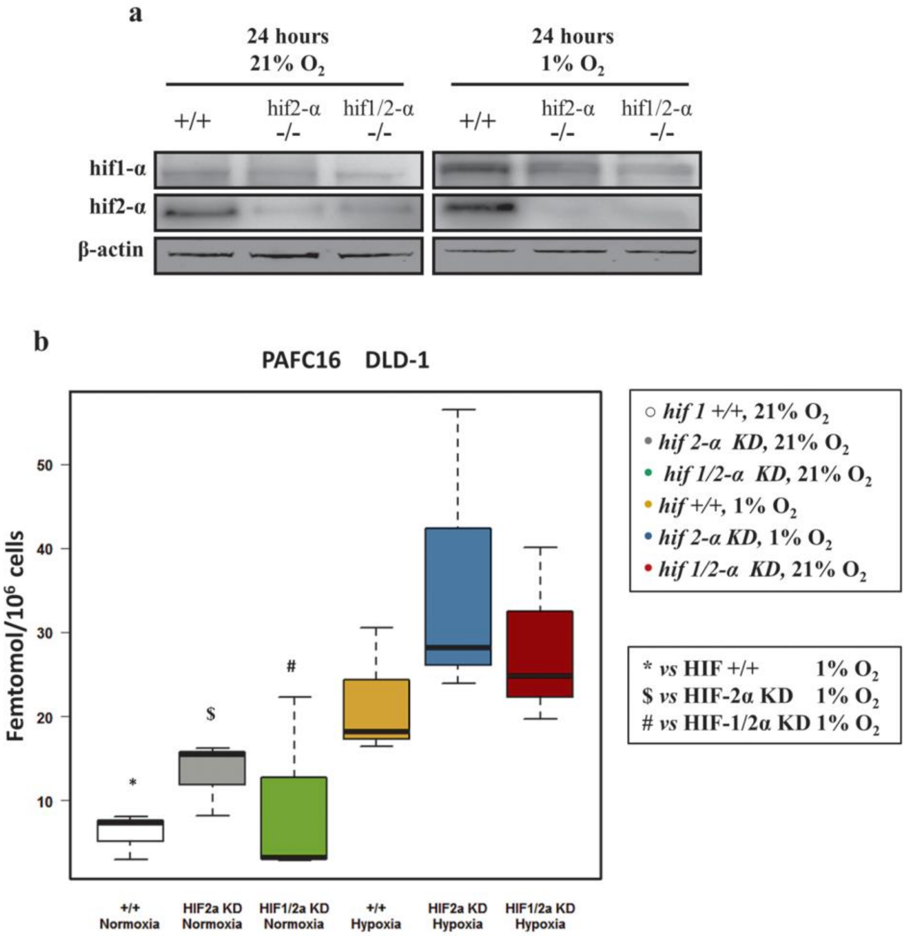

Supplemental S2: PAFC16 shows a HIF independent regulation in hypoxia: effects of HIF2α and HIF1/2α knock down.

(a) DLD-1 colon cancer cells were treated as described in the text, HIF1α and HIF2α levels, detected by western blot analysis, showed a significant knock down of both HIF2α and double HIF1/2α and hypoxic induction (n=3). **(b)** DLD-1 intracellular PAFC16 concentration reported as femtomol/10^6 cells data are shown as mean ±sd, intensities were quantified by LC/MS Q exactive (n=3).

a

femtomol / cell 21% O_2	+/+	hif1-α -/- or knock down	hif2-α knock down	hif1/2-α knock down
HCT116	4.1±0.3	3.8±3	-	-
DLD-1	6.8±4	6±2.7	-	-
DLD-1	6±2.7	-	13.3±4.5	8.9±9.1
SW122	8±4.7	5.2±0.7	-	-

b

femtomol / cell 1% O_2	+/+	hif1-α -/- or knock down	hif2-α knock down	hif1/2-α knock down
HCT116	22.4±4.1	28±10	-	-
DLD-1	22.2±15	24±3.5	-	-
DLD-1	21.7±7	-	36±17	28.2±10
SW122	34.9±21	59±45	-	-

Supplemental S3: PAFC16 concentration in HCT116, DLD-1 and SW1222 human colorectal cancer cells lines.

Comparison of the intracellular PAFC16 levels in the different cell lines utilized in the study. Data are reported as femtomol/10^6 cells and shown as mean ±sd. Intensities were quantified by LC/MS Q exactive.

a

Gene	Selection Criteria	Gene	Selection Criteria
ACAT 1	P	AGPAT2	M
ACACA	P	LPCAT2	M
ACACB	P	PPAP2B	M
FASN	P	PPAPDC1A	M
PPT1	M	PPAPDC1B	M
SCD-1	P	LPPR4	M
PLD3	P	PPAP2A	M
SREBF-1	P	PPAP2C	M
FADS1	M	LPPR5	M
FADS2	M	CHKA	M
TECR	M	CHKB	M
PECR	M	CEPT1	M
AGPAT9	M	PCYT1A	M
AGPAT6	M	PCYT1B	M
LPCAT4	M	DGKA	M
AGAPT4	M	MOGAT3	M
AGPAT1	M	MOGAT2	M
AGAPAT5	M	AGPS	M
MBOAT7	M	PLA2G7	M
LCLAT1	M	PAFAH1B2	M
MBOAT2	M	PAFAH2	M
AGPAT3	M	PAFAH1B3	M

b

	original	with annotation	with outcome
**COADREAD**	333	328	304

c

	COADREAD
**Nr of patients**	304
**Gender**	
**female**	134
**male**	170
**Age**	
**mean**	64.3
**SD**	13.1

d

	COADREAD
**median FU (years)**	0.61 (0.528 - 0.703)
**median OS (years)**	7.72 (4.76 - NA)
**5 years OS (%)**	58.1 (47.2 - 71.4)
**10 years OS (%)**	42.9 (30.4 - 60.3)

Supplemental S4: Characteristics for the selected colorectal carcinoma patients in the TCGA datasets.

(a) List of forty-four selected genes was obtained for genes related to the metabolism of the proteomics and metabolomics analysis in HCT116 cells. Selection criteria: (i) differential

abundance of enzymes observed in the *in vitro* experiments; (ii) enzymes processing hypoxia regulated metabolites observed in the *in vitro* experiments; (iii) metabolic pathway associated genes; “P” indicates proteomics experiments, “M” indicates metabolomics experiments. **(b)** Number of patients for the TCGA datasets in the preprocessing phase for survival analysis. Original denotes the number of patients with mRNA data. The “with annotation” column presents the number of patients who had annotated clinical data. Numbers in the last column represent the patients used for the survival analysis, where patients without outcome data and/or with negative or zero survival time were excluded. **(c)** Gender and age available for the relevant clinical variables. For categorical variables, the number of patients is provided. For continuous variables mean and standard deviation are provided. **(d)** Summary of clinical outcomes with 95% confidence interval provided between brackets. FU: follow-up, OS: overall survival, NA: survival time not sufficient.

Metabolite	hif1-α +/+ Normoxia	hif1-α -/- Normoxia	hif1-α +/+ Hypoxia	hif1-α -/- Hypoxia
**Acetate**	1.017e-10± 2.2e-11	1.52e-10± 5.6e-11	1.03e-10± 3.7e-11	1.81e-10± 1e-10
**Omega 3**	0.05786± 0.01	0.08791± 0.006	0.1053± 0.01	0.1436± 0.02
**Total Fatty acid**	0.1142± 0.02	0.1636± 0.009	0.1822± 0.02	0.2663± 0.05
**MUFAs**	0.0200± 0.001	0.03533± 0.002	0.0230± 0.001	0.04633± 0.004
**DiUFAs**	0.005937± 0.0003	0.01065± 0.0008	0.00952± 0.0008	0.01669± 0.002
**PUFAs**	0.005494± 0.0002	0.01109± 0.001	0.008264± 0.001	0.01708± 0.002
**Palmitate**	172920± 20400	213810± 8940	263035± 20361	323958± 86070
**Stearate**	125463± 5300	153033± 11710	206320± 17535	266002± 84112
**Oleate**	2037± 255	3040± 322	3293± 367	4990± 1711
**TAG**	0.0051± 0.0003	0.008667± 0.0005	0.0070± 0.0003	0.0124± 0.001
**Glycerol**	0.01803± 0.007	0.02603± 0.001	0.03763± 0.0008	0.0294± 0.001
**Glycerophosphate**	14.17± 7.2	10.83± 4.2	7.867± 3.2	10.67± 4.1
**Choline**	1.441e-9± 7e-10	2.387e-9± 8e-10	2.107e-9± 1e-10	7.803e-9± 1e-9
**Phosphocholine**	3.100e-8± 5e-9	3.017e-8± 2.7e-9	3.433e-8± 3.5e-9	5.917e-8 7.4e-9
**Phosphatidylcholine**	0.0390± 0.004	0.04767± 0.003	0.04433± 0.002	0.07233± 0.01
**MAG**	0.00109± 0.0003	0.001633± 0.0004	0.002133± 0.001	0.004433± 0.008
**PAF C16**	3.840e7± 2.1e7	3.437e7± 1e7	1.054e8± 6.8e7	1.147e8± 1.55e7

Supplemental S5: Raw data intensities of the assessed metabolites.

Proteins	hif1-α +/+ Normoxia	hif1-α -/- Normoxia	hif1-α +/+ Hypoxia	hif1-α -/- Hypoxia
**ACC1**	5.8e8± 1.7e8	10.2e8± 0.6e8	4.3e8± 0.2e8	7.1e8± 1e8
**ACAT1**	5.4e7± 0.3e7	3.87e7± 0.1e7	6.053e7± 0.5e7	4e7± 0.1e7
**SCD-1**	3.6e5± 0.9e5	3.5e5± 1.1e5	7.9e5± 1.4e5	11.2e5± 2.1e5
**PLD3**	15.8e7± 0.7e7	15.3e7± 6.3e7	8.5e7± 1.5e7	7e7± 1.1e7

Supplemental S6: Raw data intensities of the assessed proteins.
